# Evaluating Antibiograms To Monitor Drug Resistance

**DOI:** 10.3201/eid1108.050135

**Published:** 2005-08

**Authors:** Mohamed El-Azizi, Adnan Mushtaq, Cheryl Drake, Jerry Lawhorn, Joan Barenfanger, Steven Verhulst, Nancy Khardori

**Affiliations:** * Southern Illinois University, Springfield, Illinois, USA;; † Memorial Medical Center, Springfield, Illinois, USA

**Keywords:** Antibiotic resistance, Methicillin Resistance, Staphylococcus, Antibiogram, Presumptive Antimicrobial Therapy

## Abstract

We used hospital antibiograms to assess predominant pathogens and their patterns of in vitro antimicrobial resistance in central Illinois, USA. We found a lack of information about national guidelines for in vitro antimicrobial susceptibility testing and differences in interpretation among laboratories in the region.

A number of databases are available in the United States to monitor antimicrobial resistance at a national level (*1*). The academic and educational value of these databases is particularly useful for microbiologists and infectious disease clinicians. However, databases are unlikely to prove useful in improving antimicrobial use in the communities for a number of reasons: 1) most antimicrobial drug prescriptions in the community are written by primary care physicians, 2) most primary care physicians do not use these resources, and 3) industry-generated data are often used to highlight a particular antimicrobial drug. Even for infectious disease clinicians, national databases serve as a general guide, particularly during the initial or presumptive phase of antimicrobial therapy when culture results are not available.

For tertiary care/referral hospitals, a substantial percentage of patients are transferred from community hospitals outside the local area. Under these circumstances, resistance surveillance data from these areas would help select presumptive therapy or change existing therapy. Very often, when patients have been treated with empiric antimicrobial drugs, the culture results at the tertiary care institutions may be negative or do not detect the infecting organism. To overcome these difficulties and improve the outcome of serious infections in the referral area for our institution, we monitored resistance patterns in the region and generated a regional antibiogram, which will be shared with all participating hospitals.

## The Study

A packet was sent to the clinical microbiology laboratories of the 77 hospitals in the area. It included a letter describing the project, a questionnaire on hospital characteristics and laboratory testing methods, and a request for existing antibiograms from the most recent period for which completed data were available. We used only antibiograms from January 2001 to June 2002. From the antibiograms, the numbers of isolates tested and number of susceptible isolates were added for each antimicrobial agent from all hospitals for each region (Table A1) and for all regions combined.

The proportion of responding hospitals was 53%; all major academic centers participated. Data from 10 hospitals were excluded, 7 because the aggregated antibiograms did not include the number of isolates tested and 3 because the antibiogram data predated January 2001. Thirty-one hospitals that were included in the final analysis represented the 4 regions as follows: 16 (42%) of 38 hospitals in the central region, 6 (43%) of 14 in the west, 4 (40%) of 10 in the south, and 5 (33%) of 15 in the southwest. Of the hospitals included, 16% did not send a cumulative antibiogram but instead sent their data as a monthly report for a period from 3 months to 1 year. Our research team generated cumulative antibiograms for these hospitals.

The proposed guidelines for analyzing and presenting cumulative antimicrobial susceptibility data were published by the Clinical and Laboratory Standards Institute (formerly NCCLS) in 2002. The M39-A document provides a standardized means of data extraction for all drugs tested and outlines the most appropriate way to present the data (*2*).

In our discussions with laboratory personnel, we found that many laboratories are unaware of these guidelines, and laboratories that use the document find that adhering to all recommendations is difficult. Many laboratories lack a microbiology supervisor with insight into the clinical relevance of the results they generate. For example, a laboratory reported 4% vancomycin resistance in *Streptococcus pneumoniae*, but the laboratory staff was not able to explain this finding or recognize the clinical implications. Also 2 of the hospitals reported 2 vancomycin-intermediate *Staphylococcus aureus* in their antibiogram. However, the isolates were not available for verification, and the laboratory staff was not aware of the implications of this finding. The staff did not know that such findings should be reported to the Illinois Department of Public Health and the Centers for Disease Control and Prevention.

In all regions, *Escherichia coli* was the most commonly isolated organism, followed by *S. aureus*. Coagulase-negative staphylococci, *Pseudomonas aeruginosa*, and *Enterococcus faecalis* were among the 5 most frequently reported species (Tables A1 and A2). The 10 most frequently reported species in our study are generally comparable to those found in the SENTRY survey conducted by Pfaller et al. (*3*).

Of the *S. aureus* isolates tested in the central, west, south, and southwest regions, 27%, 53%, 34%, and 42%, respectively, were resistant to methicillin. Of the hospitals that reported speciation of enterococci, *E. faecalis* was susceptible to vancomycin at 91%–99%. The vancomycin resistance among *E. faecium* was 32%–73%. However, hospitals from the southwest area reported enterococci other than *E. faecalis* as *Enterococcus* spp. only. The unusually low susceptibility of *E. faecium* in our study may be attributed to specimen duplication.

In the central Illinois region, the susceptibility of *S. pneumoniae* to penicillin was 64%–75%, and 52%–77% of isolates were susceptible to erythromycin. The susceptibility of common gram-positive bacteria in our study appears to be lower than reported national averages (*3*). Although antibiogram surveillance and active surveillance yield comparable results (*4*), national data may not be directly comparable to our findings because national data used for comparison results from active surveillance with different reporting periods. In addition, geographic factors must be taken into consideration (*4*–*7*).

## Conclusions

In spite of expertise and resources available in the United States, the use of antimicrobial drugs in day-to-day practice is suboptimal and directly responsible for multidrug resistance in a number of common pathogens. The factor that converts antimicrobial therapy from "empiric" to "rational" is in vitro susceptibility testing and reporting. However, if these tests are either not conducted or conducted poorly, they are not useful clinically and may create a false sense that therapy is rationally guided. Given the differences and shortcomings we reported among laboratories in a region, national recommendations are either unknown or not followed. Use of expertise, cooperation, and collaboration at the regional levels may be the simplest and most useful public health measures to optimize the usefulness of diagnostic microbiology in managing infectious diseases. Antimicrobial drug use guidelines, if they are based on consistent, reproducible, and comparable data between different laboratories, will produce better outcomes. A master antibiogram for a region would allow a tertiary care institution to consider resistance patterns in hospitals referring patients and to select appropriate "presumptive" antimicrobial therapy or change drugs in nonresponding patients. We hope that the concept of "empiric antimicrobial therapy" would be changed to that of "presumptive antimicrobial therapy" based on host factors, common pathogens, and known susceptibility patterns in any given region.

This study has helped us identify serious shortcomings in susceptibility testing methods and reporting, and we hope to address these issues through a regional advisory group. Even if following all the recommendations in M39-A are not possible, the second best option may be to have all regional laboratories adhere to the same subset of recommendations. Antimicrobial resistance data generated by this approach will have better day-to-day application than will data generated by large national databases. The data will also be useful in monitoring resistance trends in a region over time and assessing the effects of interventions to reduce antimicrobial resistance. We recognize the shortcomings of the data presented in this article but believe them to be the basis for improvement at a fundamental level.

## Appendix

**Table A1:** Comparison of susceptibility results of common gram-negative bacteria to selected antimicrobial agents in 4 regions of central Illinois*

Organism/region (no. isolates tested)	% isolates susceptible (no. isolates reported)												
	AMP/SUL	CFZ	CFT	CFO	CFA	GNT	TOB	AKN	CIP	LEV	IMI/CIL	TMP/SMX	PIP
*Escherichia coli*													
West (2,600)	69 (2,600)	90 (2,600)	100 (2,347)	–	98 (1,910)	97 (2,600)	98 (2,347)	100 (2,347)	92 (978)	91 (2,369)	100 (2,600)	88 (2,347)	80 (978)
Southwest (1,024)	67 (1,006)	73 (761)	100 (1,024)	–	99 (533)	97 (1,024)	99 (1,024)	100 (906)	94 (661)	94 (754)	100 (906)	89 (1,024)	76 (1,006)
Central (11,804)	73 (7,497)	80 (9,777)	98 (7,307)	–	97 (6,175)	97 (11,683)	95 (7,271)	100 (5,615)	94 (1,063)	93 (6,689)	93 (1,364)	87 (6,570)	72 (10,371)
South (1,682)	68 (1,682)	86 (1,682)	94 (1,682)	–	98 (1,549)	97 (1,682)	99 (1,682)	100 (1,336)	91 (115)	93 (1,336)	100 (1,682)	91 (1,682)	75 (1,603)
p value	<0.0001	<0.0001	<0.0001	–	0.0020	0.9999	<0.0001	NA	0.2235	0.0049	<0.0001	<0.0001	<0.0001
*Pseudomonas aeruginosa*													
West (1,680)	–	–	–	–	88 (1,635)	78 (1,680)	95 (1,635)	94 (1,635)	73 (504)	86 (1,626)	91 (1,680)	–	92 (504)
Southwest (357)	–	–	–	–	90 (350)	80 (357)	95 (357)	96 (250)	81 (47)	68 (257)	94 (250)	–	91 (350)
Central (3,186)	–	–	–	–	85 (2,710)	77 (3,186)	91 (3,113)	95 (1,870)	67 (2,890)	60 (1,640)	89 (1,367)	–	93 (2,981)
South (591)	–	–	–	–	67 (535)	55 (591)	74 (591)	95 (522)	67 (69)	45 (522)	84 (236)	–	81 (586)
p value	–	–	–	–	<0.0001	<0.0001	<0.0001	0.0364	0.0128	<0.0001	0.0006	–	<0.0001
*Proteus mirabilis*													
West (663)	95 (663)	91 (663)	100 (625)	96 (480)	99 (568)	95 (663)	98 (625)	99 (625)	85 (480)	86 (653)	99 (66)	89 (663)	94 (290)
Southwest (294)	94 (285)	78 (264)	100 (294)	100 (155)	100 (185)	97 (294)	97 (294)	98 (185)	89 (27)	93 (194)	97 (185)	86 (294)	81 (285)
Central (2,469)	92 (1,445)	84 (1,877)	98 (1,417)	98 (867)	84 (1,423)	91 (2,469)	91 (1,760)	94 (1,421)	71 (2,009)	73 (1,489)	99 (311)	82 (1,882)	88 (2,072)
South (483)	81 (483)	73 (483)	100 (483)	100 (483)	99 (451)	97 (483)	95 (483)	100 (483)	41 (213)	77 (483)	98 (483)	79 (483)	72 (428)
p value	<0.0001	<0.0001	<0.0001	<0.0001	<0.0001	<0.0001	<0.0001	<0.0001	<0.0001	<0.0001	0.1139	<0.0001	<0.0001
*Klebsiella pneumoniae*													
West (1,236)	–	90 (1,236)	99 (1,192)	97 (395)	99 (1,109)	99 (1,236)	100 (1,335)	100 (1,161)	96 (426)	96 (1,013)	100 (1,176)	93 (1,236)	73 (426)
Southwest (296)	–	82 (222)	93 (296)	95 (116)	95 (290)	98 (290)	99 (296)	100 (190)	97 (222)	89 (164)	100 (190)	95 (290)	86 (290)
Central (3,064)	–	89 (2,507)	98 (2,048)	98 (1,505)	85 (1,660)	99 (3,064)	99 (1,974)	93 (1,604)	95 (2,606)	93 (1,523)	100 (201)	96 (2,778)	85 (2,572)
South (409)	–	95 (409)	100 (409)	99 (409)	92 (409)	97 (409)	98 (409)	100 (409)	97 (161)	97 (409)	93 (409)	98 (409)	96 (382)
p value	–	<0.0001	<0.0001	0.0246	<0.0001	0.0003	0.0002	<0.0001	0.3802	<0.0001	<0.0001	<0.0001	<0.0001
*Serratia marcescens*													
West (251)	10 (236)	–	92 (11)	95 (81)	93 (245)	98 (251)	96 (229)	98 (251)	87 (81)	94 (214)	99 (236)	95 (251)	84 (81)
Southwest (9)	22 (4)	–	89 (9)	95 (9)	95 (9)	100 (9)	99 (9)	100 (9)	100 (5)	95 (9)	100 (9)	99 (9)	92 (9)
Central (757)	26 (396)	–	95 (482)	90 (440)	97 (382)	100 (757)	98 (539)	98 (321)	80 (557)	76(343)	100 (49)	98 (552)	93 (578)
South (44)	15 (33)	–	89 (44)	97 (33)	83 (33)	93 (44)	73 (44)	100 (33)	84 (33)	100 (33)	93 (44)	93 (44)	78 (41)
p value	<0.0001	–	0.2803	0.2938	0.0006	<0.0001	<0.0001	0.8412	0.3398	<0.0001	0.0176	0.0453	<0.0001
*Enterobacter* spp.													
West (676)	–	–	80 (657)	78 (173)	79 (624)	95 (657)	96 (657)	99 (649)	94 (181)	95 (592)	100 (645)	94 (676)	81 (269)
Southwest (201)	–	–	79 (201)	95 (43)	81 (201)	100 (201)	100 (201)	100 (101)	98 (43)	92 (101)	100 (101)	96 (201)	81 (201)
Central (1,334)	–	–	78 (567)	92 (706)	83 (735)	86 (1,111)	99 (804)	100 (555)	97 (1,246)	69 (775)	100 (226)	99 (1,201)	83 (216)
South (121)	–	–	83 (96)	65 (92)	80 (92)	97 (121)	97 (121)	100 (96)	92 (64)	93 (96)	92 (121)	92 (121)	85 (112)
p value	–	–	0.6119	<0.0001	0.3167	<0.0001	<0.0001	0.0151	0.0411	<0.0001	<0.0001	<0.0001	0.6992
*Citrobacter freundii*													
West (245)	64 (245)	–	77 (233)	82 (126)	83 (245)	95 (245)	97 (233)	100 (233)	91 (126)	91 (192)	99 (233)	86 (245)	86 (126)
Southwest (38)	87 (38)	–	100 (38)	100 (38)	92 (38)	100 (38)	100 (38)	100 (24)	98 (24)	93 (24)	100 (14)	93 (14)	97 (38)
Central (578)	80 (402)	–	81 (444)	83 (332)	83 (394)	95 (578)	96 (408)	96 (302)	89 (510)	93 (238)	99 (145)	93 (517)	79 (416)
South (85)	90 (17)	–	84 (85)	80 (85)	78 (85)	93 (85)	99 (85)	100 (68)	79 (63)	79 (68)	93 (85)	85 (85)	76 (75)
p value	<0.0001	–	0.0077	0.0479	0.2716	0.4270	0.3703	0.0043	0.0544	0.0102	0.0018	0.0062	0.0136

*AMP/SUL, ampicillin/sulbactam; CFZ, cefazolin; CFT, ceftriaxone; CFO, cefotaxime; CFA, ceftazidime; GNT, gentamicin; TOB, tobramycin; AKN, amikacin; CIP, ciprofloxacin; LEV, levofloxacin; IMI/CIL, imipenem/cilastatin; TMP/SMX, trimethoprim/sulfamethoxazole; PIP, piperacillin; NA, not applicable.

**Table A2:** Comparison of susceptibility results of common gram-positive bacteria to selected antimicrobial agents in 4 regions of central Illinois*

Organism/region (no. isolates tested)	% isolates susceptible (no. isolates reported)												
	AMP	AMP/SUL	CFZ	CFT	LEV	TMP/SMX	CFO	OXA	PEN	ERY	CLN	TET	VAN
*Staphylococcus aureus*													
West (2,201)	7 (1,987)	51 (1,932)	72 (2,201)	–	64 (1,803)	97 (2,016)	–	73 (2,201)	–	51 (1,803)	70 (2,201)	93 (2,044)	100 (2,201)
Southwest 982)	12 (615)	84 (774)	81 (882)	–	50 (553)	95 (982)	–	47 (777)	–	42 (874)	54 (974)	91 (982)	100 (982)
Central (5,313)	14 (3,036)	64 (1,969)	62 (1,376)	–	62 (4,650)	99 (4,500)	–	66 (5,344)	–	48 (4,565)	65 (5,340)	96 (4,598)	100 (5,199)
South (1,582)	13 (1,582)	50 (854)	48 (1,582)	–	45 (1,313)	96 (1,582)	–	45 (219)	–	36 (1,582)	50 (269)	87 (1,363)	100 (1,007)
p value	<0.0001	<0.0001	<0.0001	–		<0.0001	–	<0.0001	–	<0.0001	<0.0001	<0.0001	NA
*Streptococcus pneumoniae*													
West (248)	–	–	–	93 (240)	99 (156)	–	84 (245)	–	67 (234)	59 (93)	–	–	100 (234)
Southwest (51)	–	–	–	84 (51)	80 (23)	–	84 (51)	–	74 (38)	77 (13)	–	–	96 (28)
Central (782)	–	–	–	82 (319)	99 (366)	–	80 (366)	–	64 (768)	64 (630)	–	–	100 (750)
South (223)	–	–	–	96 (223)	99 (121)	–	66 (29)	–	75 (16)	52 (158)	–	–	100 (86)
p value	–	–	–	<0.0001	<0.0001	–	0.0843	–	0.4417	0.0256	–	–	<0.0001
Coagulase-negative staphylococci													
West (2,066)	6 (1,868)	–	32 (2,206)	–	46 (2,066)	–	–	–	–	–	–	–	100 (2,066)
Southwest (816)	29 (729)	–	73 (816)	–	55 (816)	–	–	–	–	–	–	–	100 (816)
Central (3,583)	30 (716)	–	24 (1,125)	–	78 (1,473)	–	–	–	–	–	–	–	100 (3,583)
South (97)	8 (54)	–	44 (54)	–	64 (97)	–	–	–	–	–	–	–	100 (97)
p value	<0.0001	–	<0.0001	–	<0.0001	–	–	–	–	–	–	–	NA
*Enterococcus faecalis*													
West (803)	97 (707)	–	–	–	47 (803)	–	–	–	–	–	–	43 (803)	99 (803)
Southwest (414)	84 (414)	–	–	–	56 (136)	–	–	–	–	–	–	44 (236)	91 (414)
Central (3,797)	94 (3,152)	–	–	–	40 (2,087)	–	–	–	–	–	–	41 (2,143)	96 (3,763)
South (873)	93 (873)	–	–	–	32 (454)	–	–	–	–	–	–	40 (579)	96 (873)
p value	<0.0001	–	–	–	<0.0001	–	–	–	–	–	–	0.5635	<0.0001
*E. faecium*													
West (273)	20 (270)	–	–	–	–	–	–	–	–	–	–	40 (286)	32 (273)
Southwest (100)	22 (100)	–	–	–	–	–	–	–	–	–	–	30 (100	NV
Central (215)	22 (212)	–	–	–	–	–	–	–	–	–	–	32 (56)	55 (215)
South (419)	68 (419)	–	–	–	–	–	–	–	–	–	–	55 (125)	73 (419)
p value	<0.0001	–	–	–	–	–	–	–	–	–	–	0.0065	NA

*AMP, ampicillin; AMP/SUL, ampicillin/sulbactam; CFZ, cefazolin; CFT, ceftriaxone; LEV, levofloxacin; TMP/SMX, trimethoprim/sulfamethoxazole; CFO, cefotaxime; OXA, oxacillin; PEN, penicillin; ERY, erythromycin; CLN, clindamycin; TET, tetracycline; VAN, vancomycin; NA, not applicable; NV, not available.
